# Social Support, Resilience, and Mental Health Among Three High-Risk Groups in Hong Kong: A Mediation Analysis

**DOI:** 10.3389/ijph.2024.1606828

**Published:** 2024-04-12

**Authors:** Eliza Lai-Yi Wong, Hong Qiu, Kai-Sing Sun, Phoenix Kit-Han Mo, Angel Hor-Yan Lai, Carrie Ho-Kwan Yam, Ho-Yee Miao, Annie Wai-Ling Cheung, Eng-Kiong Yeoh

**Affiliations:** ^1^ Centre for Health Systems and Policy Research, JC School of Public Health and Primary Care, Faculty of Medicine, The Chinese University of Hong Kong, Shatin, Hong Kong SAR, China; ^2^ JC School of Public Health and Primary Care, Faculty of Medicine, The Chinese University of Hong Kong, Shatin, Hong Kong SAR, China; ^3^ Department of Applied Social Sciences, Faculty of Health and Social Sciences, The Hong Kong Polytechnic University, Kowloon, Hong Kong SAR, China

**Keywords:** anxiety, causal mediation analysis, depression, resilience, social support, vulnerable/high-risk population

## Abstract

**Objectives:**

To compare the prevalence of anxiety/depression, resilience, and social support among nurses, foreign domestic helpers (FDHs), and residents living in subdivided units (SDUs), and to examine their associations in these high-risk groups in Hong Kong during Omicron waves.

**Methods:**

We recruited 1,014 nurses, 621 FDHs, and 651 SDU residents from December 2021 to May 2022 in this cross-sectional survey. The depression, anxiety, social support, and resilience levels were measured by the validated scales. The multivariate binary logistic regression and causal mediation analysis were applied to examine the associations.

**Results:**

We observed a prevalence of 17.7% in anxiety and 21.6% in depression which were the highest in SDU residents, followed by FDHs, and lowest in nurses. Social support was associated with increased resilience levels and decreased risks of anxiety/depression. The association of social support with mental disorders was partly mediated by resilience, accounting for 30.9% and 20.9% of the total effect of social support on anxiety and depression, respectively.

**Conclusion:**

Public health strategies should target improving social support and providing resilience-promoting interventions to help reduce mental disorders in vulnerable groups.

## Introduction

Billions of people have suffered from the COVID-19 pandemic globally for more than 3 years. Besides around 764.5 million confirmed cases infected with COVID-19 and 6.9 million deaths worldwide [1], numerous mental health problems emerged during the pandemic including slightly increased anxiety and depression symptoms [2], higher prevalence of social isolation and loneliness [3], and post-traumatic stress disorder [4]. Compared with the general population, some groups of people are considered to be at higher risk of exposure to SARS-CoV-2 infection such as healthcare workers, migrant workers, children, older adults, and people living in congregate settings. There is a shortage of healthcare workers in Hong Kong, leading to their heavy workload and high stress levels. Foreign domestic workers (FDWs) account for 4.6 (%) of the total population at the end of 2021 in Hong Kong live with local families, play a crucial role in the domestic work sector and contribute an essential part of the city’s economy and society [5]. Residing in an employer’s home may result in a lack of privacy, long working hours, and challenges to maintaining relationships and social connections outside work. Meanwhile, around 92,000 households reside in subdivided units (SDUs) in Hong Kong with bad living conditions in terms of tiny and cramped living spaces, fire safety, environmental hygiene, and water seepage [6]. During the COVID-19 pandemic in the past 3 years, nurses contributing to the major portion of healthcare workers, FDWs, and people residing in the SDUs may face more challenges and worse situations [4, 7, 8]. However, the mental health problems of FDWs were underreported [9, 10] and those of people residing in the SDUs were not studied.

Evidence documented in the literature showed a considerable impact of the COVID-19 pandemic on the psychological wellbeing of frontline hospital staff [11], a decline in the mental health of nurses [4, 12], harms the physical and mental wellbeing of FDWs [13, 14], and negative impact on people living in SDUs in Hong Kong [15]. Factors associated with mental health have also been studied. Amongst the healthcare workers, frontline nurses caring for COVID-19 patients, a female, individuals with poor health conditions, and those who lived with their elderly parents at home, with low self-efficacy, received less social support and resilience were more likely to show psychological problems [4, 16, 17]. On the contrary, healthcare workers who had systemic support, adequate knowledge, and resilience were identified as protective factors against adverse mental health outcomes [11]. Resilient nurses and those who perceived higher organizational and social support were more likely to report lower anxiety related to COVID-19 [18]. Evidence of existing peer support services for improving mental health among FDWs has been reviewed [14], however, the social support and resilience of FDWs and SDU residents as vulnerable groups were underreported and not well studied [19].

We conducted the current public survey among the three high-risk groups with the aims to assess their mental health problems during the COVID-19 pandemic and to explore the risks encountered, resilience and protective factors. The current study would compare mental health problems, resilience, and social support among the three groups, and examine the association of social support with mental health problems and the potential mediation effect of resilience. We hypothesized that increasing social support may improve resilience and then mental health during the COVID pandemic.

## Methods

### Study Setting and Participants

This is a cross-sectional survey and the baseline of longitudinal surveys in three groups including nurses, foreign domestic helpers (FDHs), and residents in SDUs who show variation in risk and resilience against COVID-19 infection despite the perceived high risk of contracting the disease in their environment [20]. These three groups are chosen as they encounter significant infection risk in their environmental settings but with variation of incidence of COVID-19 observed. It is meaningful to explore their risk encountered and the resilience/protective factors to infection.

Before conducting the questionnaire surveys, we trained the interviewers and had a pilot study with in-depth interviews involving 18 nurses, 17 FDHs, and 12 residents in SDUs from August to December 2021 to inform the design of the questionnaires. We calculated the required sample size for comparing the prevalence rates among three groups. When setting the type I error of 0.05, type II error of 0.2 (statistical power of 0.8), and getting the prevalence rate of anxiety/depression at around 10% in nurses and 16%–20% in FDHs and SDU residents from the pilot study, the sample size was calculated as 197–490 for each group. This sample size was also fulfil the requirement for a mediation analysis [21].

The current survey was carried out during the fifth wave of the COVID pandemic between mid-December 2021 and early May 2022, with a convenience sample of 1,014 nurses, 621 FDHs, and 651 residents living in the SDUs participating. The fifth wave was dominated by Omicron and the incidence of confirmed COVID-19 cases was reported as 16.1% and the cumulative death rate was 122.9 per 100,000 population during the study period [22]. The nurses were recruited from the Association of Hong Kong Nursing Staff, who directly filled in the survey online in English. FDHs were recruited through the Associations of Filipino and Indonesian workers and the relevant non-governmental organizations (NGOs) such as Caritas Hong Kong which provided support services to domestic helpers. Similarly, residents in SDUs were invited through over 20 NGOs in different districts with support groups such as Baptist Oi Kwan Social Service, People Service Centre, and Mong Kok Kai Fong Association Limited Chan Hing Social Service Centre. An online survey in Chinese was conducted for the SDU residents in January 2022, and a telephone survey was arranged for those SDUs with lower education levels. An online survey using questionnaires in English and Indonesian was conducted for FDHs from January to May 2022. This study was approved by the Survey and Behavioural Ethics Committee of the Chinese University of Hong Kong (reference no. SBRE-20-581).

### Data Collection and Measurement Tools

#### Mental Health Problems

Mental health wellbeing was measured by the Patient Health Questionnaire 2-item (PHQ-2) [23] and Generalized Anxiety Disorder 2-item (GAD-2) [24]. The 2 items in PHQ-2 are “little interest or pleasure in doing things” and “feeling down, depressed, or hopeless,” while in GAD-2 are “feeling nervous, anxious or on edge” and “not being able to stop or control worrying.” The PHQ-2 and GAD-2 inquired about the frequency of depressed/anxious mood over the past 2 weeks, scoring each as 0 (“not at all”) to 3 (“nearly every day”), resulting in the score of PHQ-2 or GAD-2 ranging from 0 (lowest risk) to 6 (highest risk). Using a cut-off score of 3, participants were categorized into the high- and low-risk of depression and anxiety groups, respectively. The construct and criterion validity of the PHQ-2 and GAD-2 make them attractive measures for mental health screening [23, 24].The Cronbach’s α coefficient of the PHQ-2 and GAD-2 in the current study was 0.794 (95% CI: 0.776–0.810) and 0.827 (95% CI: 0.812–0.841), respectively, showing good internal reliability.

#### Social Support

Social support and neighborhood social capital was measured by the Multidimensional Scale of Perceived Social Support (MSPSS), which contains 12 items and 3 subscales (4 items for each) addressing the different sources of support from 1) Family, 2) Friends, and 3) Significant others [25]. The MSPSS has been demonstrated to have strong factorial and construct validity, as well as internal and test-retest reliability. Participants were asked if they received any material assistance and emotional support during the COVID-19 pandemic from three sources respectively using a 7- point Likert scoring method, with scores ranging from 1 for “low support” to 7 for “high support.” A total mean score for overall social support and three mean scores for social support from family, friends, and significant others were calculated, respectively. The Cronbach’s α coefficient of the MSPSS in the current study was 0.951 (95% CI: 0.948–0.954), and was 0.914 (0.909–0.920), 0.932 (0.927–0.936) and 0.917 (0.912–0.923) for the sub-scale of MSPSS from family, friends and significant others, respectively, showing excellent internal consistency.

#### Resilience

Resilience was measured by the abbreviated 2-item version of the Connor–Davidson Resilience Scale (CD-RISC2) [26]. The scale consists of two items—“Able to adapt when changes occur” and “Tend to bounce back after illness, injury, or other hardships”—using a 5-point Likert-type response scale from “completely incapable” (0) to “completely capable” [4]. Total scores ranged from 0 to 8, with a higher score representing a higher level of resilience. The CD-RISC2 has been demonstrated to have good test-retest reliability, convergent and discriminant validity as well as significant correlation with the overall CD-RISC score [26]; and the Chinese version of the CD-RISC2 has been shown as a reliable and valid measure of resilience assessment in the Hong Kong population [27]. We used the median value of the CD-RISC2 score as a cutoff point to identify the high (>=5) and low (<5) resilience levels. The internal consistency of the CD-RISC2 was good with Cronbach’s alpha = 0.799 (95% CI: 0.782–0.815) in the current study.

#### Social Demographical Information

Social demographical information including age group (18–34, 35–49, 50–64, ≥65 years), gender, education level (Lower secondary and below, Upper secondary, Post-secondary, Bachelor’s degree, Master’s or doctoral degree), marital status (Single, Married, Divorced/Separated/Widow), monthly family income (<HK$25,000, HK$25,000–40,000, ≥HK$40,000), employment status (Full-time, Part-time, Unemployed), chronic diseases (Yes or No), and holding religions or not were also collected during the questionnaire survey.

### Statistical Analysis

Descriptive analyses were performed to summarize the socio-demographic characteristics, mental health problems, resilience, and social support in the study samples. The Chi-square test for categorical variables and one-way ANOVA for continuous variables were used to compare the differences among the three high-risk groups.

The association of social support with resilience level and risk of anxiety or depression was examined in high-risk participants by the multivariate binary logistic regression, with social support as the predictor while adjusting the social-demographical covariates. We also fit a binary logistic regression model for anxiety or depression, with both resilience level and social support as predictors in the model while adjusting the same social-demographical covariates. The potential mediation effect of resilience level in the relationship between social support and the risk of anxiety or depression was examined by the Causal Mediation Analysis using the nonparametric bootstrapping approach to estimate the 95% confidence Intervals with the percentile method and 2000 simulations [28].

Data processing and all analyses were conducted using R version 4.1.2 (R Foundation for Statistical Computing, Vienna, Austria) with the “mediation” package for causal mediation analysis. Any two-sided *p* < 0.05 was regarded as statistically significant.

## Results

### Social-Demographics

Among the 2,286 eligible participants recruited in the survey, 1,014 were nurses, 621 were FDHs, and 651 were SDU residents. The majority of respondents were female (86.2%) and 18–49 years old (78.1%). The social-demographic characteristics of the participants in the three high-risk groups are summarized in Table 1. The proportion aged younger than 50 years old was highest among the FDH group (89.0%), followed by the SDU group (75.8%) and nurses (73.1%). In general, nurse participants were the youngest and the participants from SDU residents were the oldest in this sample. Female participants were dominant at 99.7% in FDHs, 85.8% in nurses, and 73.9% in the participants from SDU residents. Education level was highest in nurses, followed by FDHs, and lowest in the SDU residents. Nurses had the highest monthly family income, and highest proportion of being single (41.2%) followed by FDHs (23.7%) and SDU residents (7.4%). Furthermore, nurses had the highest prevalence of chronic diseases as 21.5%, followed by participants from SDU residents (20.0%) and FDHs (2.7%). Meanwhile, most of the FDHs held religion (96.3%), much greater than that of the nurses (34.6%) and SDU residents (18.6%).

**TABLE 1:** Socio-demographical characteristics among three high-risk groups* (Hong Kong, 2021-2022).

Characteristics	Total (*N* = 2,286)	Three high-risk groups			*p*-value^#^
		Nurse (*N* = 1,014)	FDH (*N* = 621)	SDU (N = 651)	
Age group^a^					<0.001
18–34 years	721 (31.5)	443 (43.7)	135 (21.7)	143 (22.0)	
35–49 years	1,066 (46.6)	298 (29.4)	418 (67.3)	350 (53.8)	
50–64 years	403 (17.6)	250 (24.7)	48 (7.7)	105 (16.1)	
≥65 years	60 (2.6)	13 (1.3)	3 (0.5)	44 (6.8)	
Gender^b^					<0.001
Man	306 (13.4)	137 (13.5)	1 (0.2)	168 (25.8)	
Woman	1970 (86.2)	870 (85.8)	619 (99.7)	481 (73.9)	
Education^c^					<0.001
Lower secondary and below	460 (20.1)	2 (0.2)	119 (19.2)	339 (52.1)	
Upper secondary	386 (16.9)	58 (5.7)	131 (21.1)	197 (30.3)	
Post-secondary	497 (21.7)	216 (21.3)	217 (34.9)	64 (9.8)	
Bachelor’s degree	547 (23.9)	439 (43.3)	79 (12.7)	29 (4.5)	
Master’s or doctoral degree	281 (12.3)	281 (27.7)	-	-	
Marital status^d^					<0.001
Single	613 (26.8)	418 (41.2)	147 (23.7)	48 (7.4)	
Married	1,239 (54.2)	526 (51.9)	318 (51.2)	395 (60.7)	
Divorced/Separated/Widow	338 (14.8)	31 (3.1)	124 (20.0)	183 (28.1)	
Monthly family income^e^					<0.001
< HK$25,000	1,258 (55.0)	84 (8.3)	621 (100.0)	553 (84.9)	
25,000 ∼ < 40,000	125 (5.5)	104 (10.2)	-	21 (3.3)	
≥ HK$40,000	656 (28.7)	652 (64.3)	-	4 (0.7)	
Employment status					<0.001
Full-time	1,578 (69.0)	856 (84.4)	621 (100.0)	101 (15.5)	
Part-time	518 (22.7)	125 (12.3)	-	393 (60.4)	
Unemployed or no answer	190 (8.3)	33 (3.3)	-	157 (24.1)	
Chronic diseases^f^					<0.001
No	1803 (78.9)	744 (73.4)	583 (93.9)	476 (73.1)	
Yes	365 (16.0)	218 (21.5)	17 (2.7)	130 (20.0)	
Religions^g^					<0.001
No	1,148 (50.2)	632 (62.3)	9 (1.4)	507 (77.9)	
Yes	1,070 (46.8)	351 (34.6)	598 (96.3)	121 (18.6)	

*: Data are presented as the number of participants in each category (N), together with the column percentage (%) for categorical variables. ^#^: *p*-values are got from the Chi-square test for categorical variables.

^a^: 36, ^b^: 10, ^c^: 115, ^d^: 96, ^e^: 247, ^f^: 118, ^g^: 68 participants refused to answer the question and were re-coded as a specific category in the analyses. Abbreviations: FDH, foreign domestic helper; SDU, resident in subdivided units.

### Social Support, Resilience, and Mental Health

The mental health problems were most severe in the SDU residents, followed by FDHs and nurses. The prevalence of anxiety and depression were the highest in SDU residents (34.4% and 37.9%, respectively), higher than those of FDHs (13.0% and 21.4%), and lowest in nurses (9.8% and 11.1%) (Table 2). The resilience level was highest in FDHs with a mean score of 6.3, followed by nurses (5.1), and the lowest in SDU residents (4.6). Social support showed the highest mean score of 5.2 in nurses, followed by FDHs with a mean score of 5.1, and lowest in SDU residents with a mean score of 4.5. The subscales of social support from family, friends, and significant others showed similar patterns among the three high-risk groups (Table 2).

**TABLE 2:** Mental health problem, resilience, and social support among three high-risk groups * (Hong Kong, 2021-2022).

Characteristics	Total (N = 2,286)	Three high-risk groups			*p*-value^#^
		Nurse (N = 1,014)	FDH (N = 621)	SDU (N = 651)	
Anxiety					<0.001
GAD-2 < 3	1882 (82.3)	915 (90.2)	540 (87.0)	427 (65.6)	
GAD-2 ≥ 3	404 (17.7)	99 (9.8)	81 (13.0)	224 (34.4)	
Depression					<0.001
PHQ-2 < 3	1793 (78.4)	901 (88.9)	488 (78.6)	404 (62.1)	
PHQ-2 ≥ 3	493 (21.6)	113 (11.1)	133 (21.4)	247 (37.9)	
Resilience (CD-RISC2)	5.3 ± 1.7	5.1 ± 1.4	6.3 ± 1.8	4.6 ± 1.7	<0.001
CD-RISC2 < 5	822 (36.0)	359 (35.4)	122 (19.6)	341 (52.4)	<0.001
CD-RISC2 ≥ 5	1,464 (64.0)	655 (64.6)	499 (80.4)	310 (47.6)	
Social support	5.0 ± 1.2	5.2 ± 1.1	5.1 ± 1.1	4.5 ± 1.3	<0.001
From family	5.0 ± 1.4	5.1 ± 1.3	5.3 ± 1.3	4.5 ± 1.5	<0.001
From friends	4.9 ± 1.3	5.2 ± 1.1	5.0 ± 1.2	4.3 ± 1.5	<0.001
From significant others	5.0 ± 1.3	5.2 ± 1.2	5.0 ± 1.2	4.5 ± 1.4	<0.001

*: Data are presented as the number of participants in each category (N), together with the column percentage (%) for categorical variables and Mean ± SD, for continuous variables. ^#^: *p*-values are got from the Chi-square test for categorical variables and one-way ANOVA, for continuous variables. Abbreviations: FDH, foreign domestic helper; SDU, Resident in Subdivided Units. GAD-2, generalized anxiety disorder 2-item; PHQ-2, patient health questionnaire 2-item; CD-RISC2, connor–davidson resilience scale 2-item.

### Association of Social Support With Resilience and Mental Health

After adjusting the socio-demographical characteristics among all participants, we observed the association of social support with increased resilience levels and decreased risks of mental health problems (Table 3). A score increment in social support was associated with an OR of 1.47 (95% CI: 1.35–1.60) for high resilience level and decreased odds of anxiety and depression with the same ORs of 0.82 (95% CI: 0.75–0.90). Social support from family, friends, and significant others showed similar effect estimates. Compared with nurses, FDHs had significantly higher resilience levels with an OR of 3.44 (95% CI: 2.14–5.52) while participants from SDU residents had greater odds of anxiety and depression with an OR of 2.72 (95% CI: 1.57–4.72) and 2.53 (95% CI: 1.51–4.23), respectively. Among the social-demographical factors adjusted in the current regression models, participants with middle ages and higher monthly family income showed a decreased risk of having mental health problems. Participants with chronic diseases tended to have a higher risk of depression, with an OR of 1.51 (1.10–2.07).

**TABLE 3:** The association of social support with high resilience level and mental health problems* (N = 2,286) (Hong Kong, 2021-2022).

Associated factors	High resilience level (CD-RISC2≥5) OR (95% CI)	*p*-value	Anxiety OR (95% CI)	*p*-value	Depression OR (95% CI)	*p*-value
**Social support** *, per 1 score increase*	**1.47 (1.35, 1.60)**	**< 0.01**	**0.82 (0.75, 0.90)**	**< 0.01**	**0.82 (0.75, 0.90)**	**< 0.01**
From family	**1.32 (1.23, 1.41)**	**< 0.01**	**0.82 (0.76, 0.89)**	**< 0.01**	**0.85 (0.78, 0.92)**	**< 0.01**
From friends	**1.37 (1.27, 1.48)**	**< 0.01**	**0.87 (0.80, 0.95)**	**< 0.01**	**0.87 (0.80, 0.94)**	**< 0.01**
From significant others	**1.38 (1.28, 1.49)**	**< 0.01**	**0.87 (0.80, 0.95)**	**< 0.01**	**0.86 (0.79, 0.93)**	**< 0.01**
**Group**						
Nurses	1.00		1.00		1.00	
Foreign Domestic Helpers	**3.44 (2.14, 5.52)**	**< 0.01**	0.80 (0.44, 1.45)	0.47	1.31 (0.76, 2.28)	0.33
Residents in Subdivided Units	0.84 (0.54, 1.31	0.45	**2.72 (1.57, 4.72)**	**< 0.01**	**2.53 (1.51, 4.23)**	**< 0.01**
**Age group** ^**a**^						
18–34 years	1.00		1.00		1.00	
35–49 years	1.07 (0.85, 1.35)	0.57	1.13 (0.85, 1.51)	0.40	0.83 (0.64, 1.08)	0.17
50–64 years	**1.55 (1.15, 2.10)**	**< 0.01**	**0.57 (0.37, 0.86)**	**0.01**	**0.46 (0.31, 0.67)**	**0.01**
≥65 years	1.43 (0.78, 2.62)	0.24	0.82 (0.42, 1.63)	0.57	**0.50 (0.26, 0.98)**	**0.04**
**Gender** ^**b**^ Female vs. Male	**0.72 (0.55, 0.96)**	**0.02**	1.36 (0.95, 1.94)	0.09	1.21 (0.86, 1.69)	0.27
**Education level** ^**c**^						
Lower secondary and below	1.00		1.00		1.00	
Upper secondary	1.28 (0.94, 1.74)	0.12	0.87 (0.62, 1.22)	0.42	0.95 (0.69, 1.29)	0.73
Post-secondary	1.13 (0.80, 1.59)	0.49	1.15 (0.79, 1.69)	0.47	1.06 (0.74, 1.51)	0.75
Bachelor’s degree	**1.61 (1.10, 2.35)**	**0.01**	0.75 (0.47, 1.20)	0.24	0.84 (0.55, 1.28)	0.41
Master’s or doctoral degree	**1.74 (1.11, 2.72)**	**0.02**	0.87 (0.47, 1.60)	0.66	0.88 (0.49, 1.56)	0.65
**Marital status** ^**d**^						
Never married	1.00		1.00		1.00	
Married	0.94 (0.74, 1.19)	0.61	0.80 (0.58, 1.10)	0.17	0.99 (0.74, 1.32)	0.94
Divorced/Separated/Widow	1.14 (0.81, 1.62)	0.45	0.88 (0.59, 1.33)	0.56	1.31 (0.90, 1.91)	0.56
**Monthly family income** ^**e**^						
< HK$25,000	1.00		1.00		1.00	
25,000 ∼ < 40,000	1.21 (0.75, 1.95)	0.44	0.68 (0.36, 1.28)	0.23	0.78 (0.44, 1.41)	0.41
≥ HK$40,000	1.14 (0.77, 1.68)	0.51	0.64 (0.38, 1.08)	0.09	**0.59 (0.36, 0.97)**	**0.04**
**Employment status**						
Full-time	1.00		1.00		1.00	
Part-time	1.01 (0.74, 1.38)	0.96	1.11 (0.74, 1.66)	0.60	1.11 (0.76, 1.62)	0.60
Unemployed or no answer	1.21 (0.81, 1.83)	0.35	1.15 (0.71, 1.85)	0.58	1.30 (0.82, 2.05)	0.27
**Having chronic diseases** ^**f**^	0.85 (0.65, 1.11)	0.24	1.22 (0.87, 1.70)	0.26	**1.51 (1.10, 2.07)**	**0.01**
**Holding Religions** ^**g**^	1.05 (0.83, 1.33)	0.66	0.95 (0.70, 1.29)	0.74	1.00 (0.75, 1.35)	0.98

*: Associations were presented as OR (95% CI) and estimated from the multivariate binary logistic regression. A high resilience level was defined as a CD-RISC2 score ≥ 5, the median score. ^a^: 36, ^b^: 10, ^c^: 115, ^d^: 96, ^e^: 247, ^f^: 118, ^g^: 68 participants refused to answer the question and were re-coded as a specific category in the analyses. ORs for the sociodemographic factors were estimated with total social support in the model. ORs for social support from three sources were estimated one by one with adjustment for the sociodemographic factors. Highlighted bold values: *p*-value <0.05.

### Causal Mediation Analysis

When including both social support and resilience in the model simultaneously (Table 4), the protective effect estimates of social support on the risks of anxiety and depression remained but decreased a bit to an OR of 0.87, while the high resilience level was also associated with the decreased odds of anxiety and depression, with an OR of 0.43 (95% CI: 0.34–0.55) and 0.47 (95% CI: 0.38–0.59), respectively. Results from these regression models suggested that the negative association of social support with anxiety and depression may partly be mediated by resilience. Then we applied causal mediation analysis to examine the proportion mediated by resilience in the relationship between social support and mental health.

**TABLE 4:** The association of social support and high resilience level with mental health problems (N = 2,286) (Hong Kong, 2021-2022).

Associated factors	Anxiety OR (95% CI)^a^	*p*-value	Depression OR (95% CI)^a^	*p*-value
**Social support** *, per 1 score increase*	0.87 (0.79, 0.96)	0.01	0.87 (0.79, 0.95)	<0.01
**Resilience** ^b^				
Low resilience level	1.00		1.00	
High resilience level	0.43 (0.34, 0.55)	<0.01	0.47 (0.38, 0.59)	<0.01

^a^ OR (95%CI) was estimated from the multivariate binary logistic regression, adjusting for the same person-specific characteristics as those in Table 3 as potential confounders.

^b^ Resilience was measured by the CD-RISC2 scale and categorized into low and high levels using the median score of 5 as the cutoff point.

**FIGURE 1:**
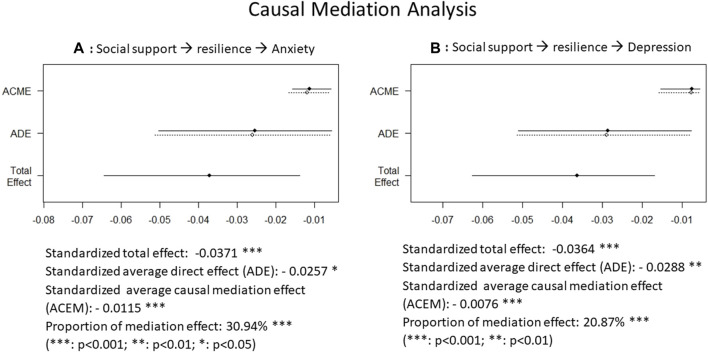
Causal mediation analysis showing the negative association of social support with anxiety and depression, partly mediated by resilience (Hong Kong, 2021-2022). **(A)** the association of social support with anxiety, mediated by resilience; **(B)** the association of social support with depression, mediated by resilience.

The results of the mediation analysis showed a standardized total effect of −0.0371 (*p* < 0.001) of social support on anxiety, a standardized average direct effect (ADE) of −0.0257 (*p* = 0.014), and a standardized average causal mediation effect (ACME) of −0.0115 (*p* < 0.001) which accounted for 30.9% (*p* < 0.001) of the total effect (Figure 1A). Similarly, a standardized total effect of −0.0364 (*p* < 0.001) of social support on depression contained a standardized ADE of −0.0288 (*p* = 0.004) and a standardized ACME of −0.0076 (*p* < 0.001) which accounted for 20.9% (*p* < 0.001) of the total effect (Figure 1B).

## Discussion

In this survey of high-risk groups during the COVID-19 pandemic, we observed a prevalence of 17.7% in anxiety and 21.6% in depression which were the highest in the SDU residents, followed by FDHs, and lowest in nurses. Social supports were highest among nurses, followed by FDHs, and lowest among the SDU residents. The resilience level was highest in FDHs, followed by nurses, and lowest in SDU residents. Social support was associated with increased resilience levels and decreased risks of mental health problems. Causal mediation analysis demonstrated that the association of social support with mental health was partly mediated by resilience, accounting for 30.9% and 20.9% of the total effect of social support on anxiety and depression, respectively.

### Compared With the Evidence in the Literature

We identified the mental health problems in the high-risk groups in Hong Kong: The observed prevalence of anxiety and depression in the current study were the highest in SDU residents (34.4% and 37.9%, respectively), higher than those of FDHs (13.0% and 21.4%), and lowest in nurses (9.8% and 11.1%). Such prevalence rates among nurses in Hong Kong were lower than the pooled 32% of anxiety and also 32% of depression generated from 25 to 17 cross-sectional studies among nurses across the globe, respectively, in a systematic review and meta-analysis in the first year of the COVID-19 pandemic [4]. Such depression prevalence in the current FDWs sample was also a bit lower than the 25.2% of mild-to-extremely-severe depression level in a survey of 105 Filipino foreign domestic helpers conducted in 2017 [10]. However, the mental health problems of those living in inappropriate housing in Hong Kong and worldwide are understudied [15]. In brief, our findings indicated that the prevalence of mental health problems varied across high-risk populations including nurses, migrant workers, and residents from inappropriate housing during the period of the COVID-19 pandemic. This observation aligns with previous studies that have observed the heterogeneity in mental health problems among different populations or during different time periods [2, 4, 29, 30].

We observed the protective effect of social support on increased resilience levels and decreased risks of anxiety and depression, consistent with the factors identified in the literature that were associated with mental health problems. For example, amongst the healthcare workers, frontline nurses, women, individuals with poor health condition, those who lived with their elderly parents at home, received less social support, and with a negative stress-coping style were more likely to show psychological problems [16, 17]. A systematic review and meta-analysis evidenced a decline in the mental health of nurses across the globe during COVID-19 and found the significant risk factors for mental ailments included caring for COVID-19 patients, being a female, low self-efficacy, low resilience, less social support and having physical symptoms [4]. Systemic support, adequate knowledge and resilience were factors protecting against adverse mental health outcomes imposed by the impact of COVID-19 among the healthcare workers [11]. One of our previous survey among 3,048 eligible HCWs in Hong Kong, Nepal, Vietnam, and Taiwan from May 2021 to July 2022 also supported that besides older age, part-time work type, higher education level, participants with better organizational supports and fewer COVID-specific worries were associated with higher resilience [31]. Resilient nurses and those who perceived higher organizational and social support were more likely to report lower anxiety related to COVID-19 [18]. Resilience and self-perceived social support were inversely associated with mental health problems (psychological distress, depression symptoms, and death thoughts) among healthcare workers in Spain, after adjusting for potential sources of confounding [32].

Furthermore, we observed the protective effect of social support on decreased risk of anxiety and depression may partly be mediated by resilience, consistent with a few evidences of such mediate role of resilience on the mental health disorders that were documented in the literature. In a cross-sectional survey of the psychological status of nurses during the COVID-19 epidemic period in Fujian China, the social support and psychological resilience were found to mediate the association between coping and mental health disorder [33]. A structural equation modeling (SEM) study found that resilience could partially mediate the effect of social support on mental health among 1,472 healthcare workers from Jiangsu Province, China during the peak period of the COVID-19 outbreak [34]. Resilience was also found to mediate 1) the perceived risk during COVID-19 pandemic or the COVID-related work stress and mental health problems among healthcare professionals [35, 36]; 2) the association of nurses’ experiences of skin lesions with anxiety and depression [37]; 3) the relationship between the fear of getting infected by COVID-19 and depression/anxiety/anger [38]; and 4) the relationships between depression/anger symptoms and cognitive failures in a large Italian sample during quarantine or self-isolation for COVID-19 [39]. Most of the previous studies on the mediation role of resilience on mental health problems were conducted among healthcare workers, we added evidence to the literature by expanding the study population to migrant workers and those living in inappropriate housing.

### Strengths and Limitations

This is the first study up to date to explore and compare the mental health problems, social supports, and resilience levels among the high-risk groups during the quick spread of the Omicron pandemic in early 2022, taking nurses, FDHs, and SDU residents into account simultaneously. We added to the literature the association between social support and mental health problems which was partly mediated by resilience level, in the high-risk groups with the diversity of the participants in social demographic characteristics. Meanwhile, some limitations should be noted. First, the cross-sectional study design could not uncover the casual relationships but only the associations, although the causal mediation analysis was applied. Further study with follow-up surveys in a longitudinal design may help to detect the causal effect. Second, there was potential selection bias as we employed a convenience sampling approach and the response rate was low. To enhance the generalization of findings, we recruit the study participants through the representative organization reaching the majority of the target population including the Association of Hong Kong Nursing Staff, the Associations of Filipino and Indonesian workers, and the key NGOs scattered in Hong Kong providing support to SUD residents, respectively. The Nurse Association sent emails to 14,950 members, and 1,014 nurses participated and finished the survey with a response rate of 6.8%. We did not have exact response rates for FDHs and SDU residents. The reasons for refusal were common such as no time or no interest to join the survey. Third, mediation analyses are subject to some strong assumptions, such as no unmeasured confounding, no measurement error, and no interaction between the mediator and the exposure [40]. The potential violations of these assumptions may distort the interpretation of the results of mediation. Fourth, the prevalence rates of depression/anxiety were screened by the PHQ-2 and GAD-2, respectively, which may have acceptable but relatively low sensitivity and specificity [41] and we should have caution in the interpretation of the prevalence rates. Finally, the prevalence rates of depression/anxiety observed among the high-risk groups may vary in the general population and change over time: they were higher than that in the general population and getting lower than those studied at the beginning of the COVID-19 pandemic. Therefore, the prevalence rate observed from the current study may not be generalized to other places with different study populations, pandemic waves, and policy supports.

### Conclusion and Implications

Within the high-risk groups during the COVID-19 Omicron waves in Hong Kong, our study revealed the protective association of social support with decreased risk of anxiety and depression which may partly be mediated by resilience. This is the first study to compare three high environmental risk groups during the COVID-19 pandemic. The findings have important implications for public health intervention and policy development, highlighting the needs to enhance social support and improve resilience during infectious disease outbreaks. Public health strategies should target improving social support and providing resilience-promoting interventions to help reduce mental health problems, particularly in vulnerable groups during the pandemic.

## Data Availability

The dataset generated and analyzed during this study is available from the corresponding authors upon reasonable request.

## Abbreviations

CD-RISC2, connor–davidson resilience scale 2-item; CI, confidence interval; COVID-19, coronavirus disease 2019; FDH, foreign domestic helper; FDW, foreign domestic worker; GAD-2, generalized anxiety disorder 2-item; PHQ-2, patient health questionnaire 2-item; MSPSS, multidimensional scale of perceived social support; NGO, non-governmental organization; OR, odds ratio; SARS-CoV-2, severe acute respiratory syndrome coronavirus 2; SDU, subdivided units.
